# Three Gorges Dam: Impact of Water Level Changes on the Density of Schistosome-Transmitting Snail *Oncomelania hupensis* in Dongting Lake Area, China

**DOI:** 10.1371/journal.pntd.0003882

**Published:** 2015-06-26

**Authors:** Jin-Yi Wu, Yi-Biao Zhou, Yue Chen, Song Liang, Lin-Han Li, Sheng-Bang Zheng, Shao-ping Zhu, Guang-Hui Ren, Xiu-Xia Song, Qing-Wu Jiang

**Affiliations:** 1 Department of Epidemiology, School of Public Health, Fudan University, Shanghai, China; 2 Key Laboratory of Public Health Safety, Ministry of Education, Fudan University, Shanghai, China; 3 Center for Tropical Disease Research, Fudan University, Shanghai, China; 4 School of Epidemiology, Public Health and Preventive Medicine, Faculty of Medicine, University of Ottawa, Ottawa, Ontario, Canada; 5 Department of Environmental and Global Health, College of Public Health and Health Professions, University of Florida, Gainesville, Florida, United States of America; 6 Emerging Pathogens Institute, University of Florida, Gainesville, Florida, United States of America; 7 Anxiang Office of Leading Group for Schistosomiasis Control, Changde, Hunan Province, China; 8 Hunan Institute for Schistosomiasis Control, Yueyang, Hunan Province, China; George Washington University School of Medicine and Health Sciences, UNITED STATES

## Abstract

**Background:**

Schistosomiasis remains an important public health issue in China and worldwide. *Oncomelania hupensis* is the unique intermediate host of *schistosoma japonicum*, and its change influences the distribution of *S*. *japonica*. The Three Gorges Dam (TGD) has substantially changed the ecology and environment in the Dongting Lake region. This study investigated the impact of water level and elevation on the survival and habitat of the snails.

**Methods:**

Data were collected for 16 bottomlands around 4 hydrological stations, which included water, density of living snails (form the Anxiang Station for Schistosomiasis Control) and elevation (from Google Earth). Based on the elevation, sixteen bottomlands were divided into 3 groups. ARIMA models were built to predict the density of living snails in different elevation areas.

**Results:**

Before closure of TGD, 7 out of 9 years had a water level beyond the warning level at least once at Anxiang hydrological station, compared with only 3 out of 10 years after closure of TGD. There were two severe droughts that happened in 2006 and 2011, with much fewer number of flooding per year compared with other study years. Overall, there was a correlation between water level changing and density of living snails variation in all the elevations areas. The density of living snails in all elevations areas was decreasing after the TGD was built. The relationship between number of flooding per year and the density of living snails was more pronounced in the medium and high elevation areas; the density of living snails kept decreasing from 2003 to 2014. In low elevation area however, the density of living snails decreased after 2003 first and turned to increase after 2011. Our ARIMA prediction models indicated that the snails would not disappear in the Dongting Lake region in the next 7 years. In the low elevation area, the density of living snails would increase slightly, and then stabilize after the year 2017. In the medium elevation region, the change of the density of living snails would be more obvious and would increase till the year 2020. In the high elevation area, the density of living snails would remain stable after the year 2015.

**Conclusion:**

The TGD influenced water levels and reduced the risk of flooding and the density of living snails in the study region. Based on our prediction models, the density of living snails in all elevations tends to be stabilized. Control of *S*. *japonica* would continue to be an important task in the study area in the coming decade.

## Author Summary

*Oncomelania hupensis*, an amphibious animal, is the unique intermediate host of *schistosoma japonicum*. Three Gorges Dam (TGD) is a tremendous hydrological project, and it influences the survival of animals downstream. It is studied for several reasons. First, schistosomiasis is still a world-wide parasitic disease which needs to be prevented and controlled. Second, TGD causes the change of water level, and it will impact the existence of snails, but how TGD causes the change of snails and even the epidemics of schistosomiasis is not revealed. In this study, the authors explore the association between TGD and snails. The time-series data contains over 10-year water level downstream TGD and 10-year density of living snails downstream TGD in Dongting Lake Region. These can help to find out the relationship between the change of water level and the change of density of living snails. After this exploration, we attempt to predict the density of living snails 7years later using ARIMA model.

## Introduction

Schistosomiasis remains a serious public health problem worldwide, affecting more than 200 million people in approximately 76 countries with a loss of 1.53 million disability-adjusted life years (DALYs) [1]. *Schistosomiasis japonica (S*. *japonica)* is distributed in 12 provinces in China and 11.6 million people have been infected since 1949 [2–4]. *S*. *japonica* causes the most hazardous schistosomiasis, and is difficult to prevent and treat [5, 6]. After continued implementations of comprehensive control measures from the mid-1950s to 1980s, endemic regions were circumscribed in certain core areas in China, especially in the Dongting Lake region at the middle reaches of the Yangtze River [7–11]. Most of these core areas are in the downstream of Three Gorges Dam (TGD). The TGD is a world-class water conservancy project. It began to impound water and sediment discharge in 2003, and is one of several tremendous engineering projects transforming China’s ecology and natural environment. The construction and the operation of TGD have obviously affected the ecological environment. The water level of TGD in 2003 was 135m and it reached 160m in 2011. It is believed that the TGD project reduces the frequency of major flooding in the downstream areas from once every ten years to once every 100 years; however it threatens the living of aquatic animals in the Yangtze River including the river dolphin, or baiji, and finless porpoise, or jiangzhu [12]. Similarly the TGD project can also influence the survival of *O*. *hupensis*, the unique intermediate host of *Schistosoma japonicum* [13, 14].

The TGD has been completed for over 10 years, and its impact on the transmission of *S*. *japonica* has to be evaluated [15, 16]. This study aimed to determine the impact of changes in water level and number of flooding per year on the snail (*O*. *hupensis)* density, and to predict the changes in the density of living snails in the Dongting Lake region using the autoregressive integrated moving average (ARIMA) model, which combines the advantages of autoregressive (AR) model and moving average (MA) model.

## Methods

### 1. Study area and sampling

**Fig 1:**
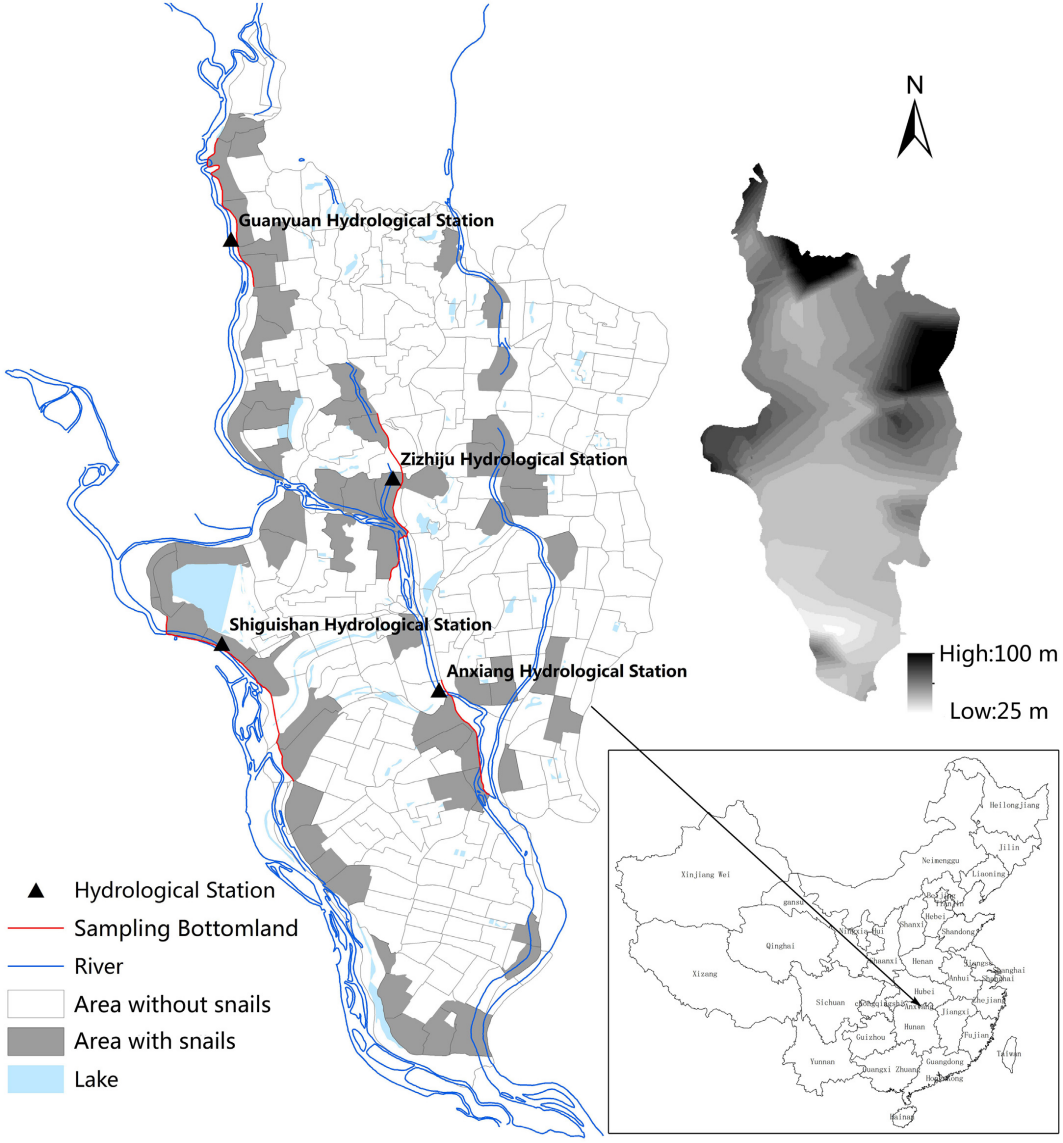
Map of sampling area and hydrological station in Anxiang county, Hunan province, People’s Republic of China.

This study was conducted in the 16 bottomlands near four hydrological stations in Anxiang County of the Dongting Lake region (Fig 1). These bottomlands are outside the embankment. The Dongting Lake is located at 28°30′–30°20′N and 111°40′–113°40′E in the northeastern part of Hunan Province and covers a water surface area of 2,681 km^2^, and plays an important role in regulating the amount of water in the Yangtze River [17]. It is a typical *S*. *japonica* endemic area with an at-risk population of about 429,000 people in marshland and lake regions [18]. Anxiang County is located in the Dongting Lake area and at the downstream of the TGD. There are 4 hydrological stations located in Anxiang County and for each hydrological station we selected 4 bottomlands as our study fields (Fig 1): 1) Guanyuan hydrological station—Qinglong, Dongbao, Nanyang and Yangshutan; 2) Zizhiju hydrological station—Yandoukou, Dongdi, Yongheyuan, Yifenju; 3) Shiguishan hydrological station—Qiangkou, Huangjiatai, Fuxing, Wuyang; and 4) Anxiang hydrological station—Yucheng, Liujiao, Zhulin, Anxiang. A total of sixteen bottomlands were divided into 3 groups according to three elevation: Low (<33m), medium (33 to 35m) and high (>35 m).

### 2. Data sources

The routine data of snails which could be obtained and used was snail density and snail area. The change in snail areas was not large in this study field and period. The density of living snails is an important index reflecting the survival and reproduction of snails. Thus the density of living snails was used to assess the impact of TGD on snails. The Anxiang Station for Schistosomiasis Control provided data of density of living snails in every bottomland of Anxiang County for the period from 2004 to 2014. Snail surveys were conducted in each spring and were implemented using a traditional method of random quadrant sampling (0.11 m^2^-sized frames, 20 m apart between frames) [19].

**Fig 2:**
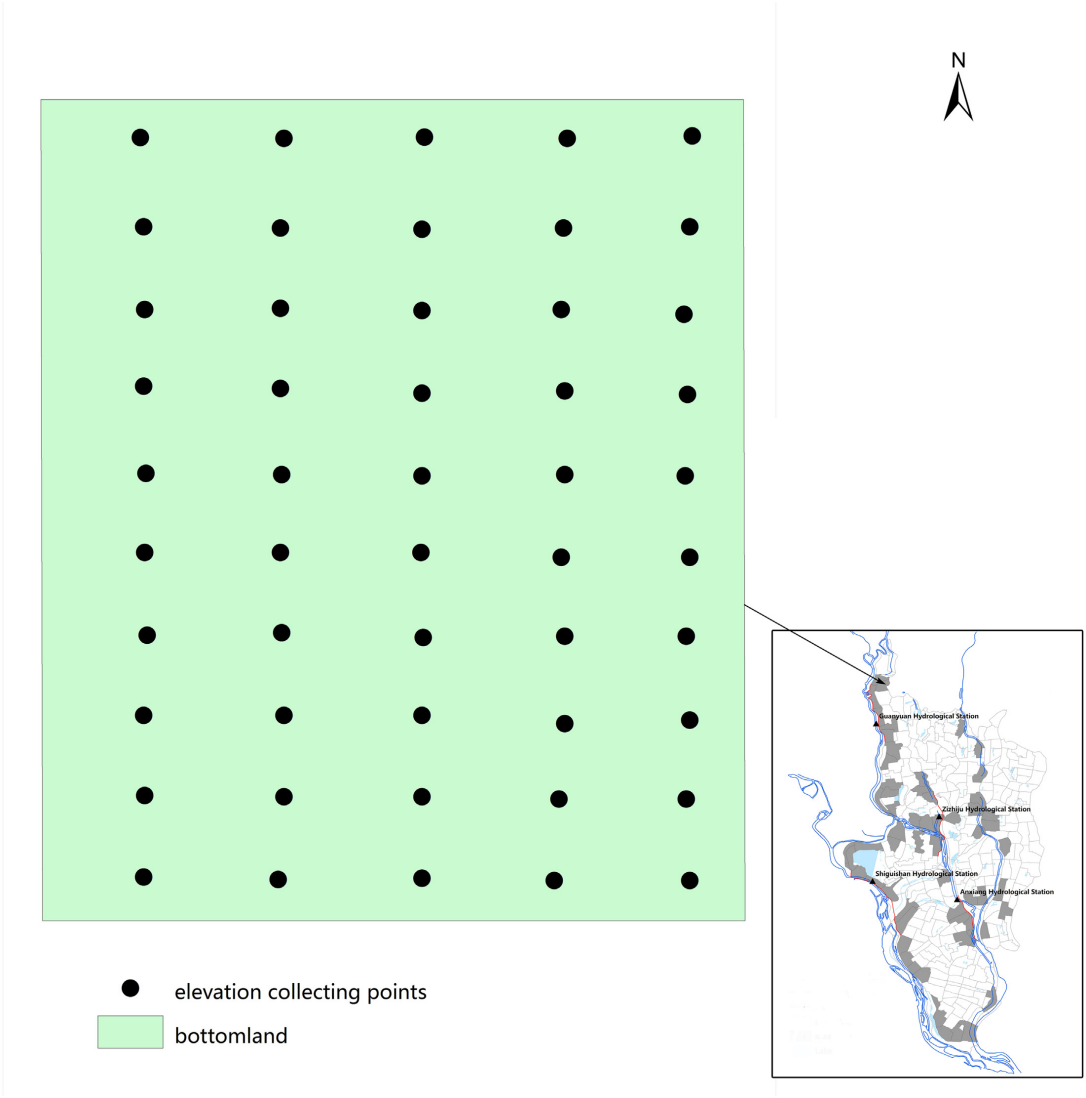
Sketch of collecting points in a bottomland in Anxiang county, Hunan province, People’s Republic of China.

We downloaded data of water levels from the Hunan Flood Prevention Information System, including daily water levels at 8:00 am at the 4 hydrological stations from 1995 to 2013. The elevation for the 4 hydrological stations was 33m for the Guanyuan station, 31m for the Zizhiju and Anxiang stations, and 30m for the Shiguishan station. Data of elevation were collected through the Google Earth (Google Ltd, USA, http://www.google.com/earth/) by sampling 50 points (5*10, 200 m apart between points) for each bottomland (Fig 2). A weighted average elevation was calculated for each bottomland.

### 3. Analysis

We calculated number of flooding per year, which is calculated in the basis of the difference between the daily water level at each hydrological station and the weighted average elevation of the bottomland near the station. If the difference was greater than 0, the bottomland around the hydrological station was considered to be flooded.

We used the density of living snails time-series data to fit an optimum ARIMA model and predict the density of living snails in the future. ARIMA model is a component of the Box-Jenkins approach to time-series modeling [20–22]. An ARIMA model is derived by combining the three techniques: autoregressive modeling (AR), moving average modeling (MA) and differencing. They are presented as ARIMA(p,d,q), in which p and q represent the orders of AR and MA models, respectively, and d denotes the order of differencing. In ARIMA models, we assume a stationary time series, which means that the data vary around a constant mean and variance over time [23–25]. Nonstationary time series variables can be converted into stationary ones. An ARIMA(p,d,q) model can be written as [26, 27]:

y't=c+ϕ1y’t-1+…+ϕpy’t-p-θ1zt-1-…-θ1zt-1+zt

where c is a constant, y’t = yt-yt-1 represents the differenced series, y’t-p are lagged value and zt is a white noise process.

The ARIMA modeling procedure consists of three iterative steps: selecting a candidate model, estimating the model and performing diagnostic tests and forecasting.

Before fitting an ARIMA model, selecting a candidate model is the process of identifying randomness, stationarity and seasonality using the autocorrelation functions (ACF) and partial autocorrelation functions (PACF). If data do not meet these requirements, a transformation of data should be implemented [28]. ACF is a statistical tool that measures whether an earlier value in the series has some relation to a later value. PACF captures the amount of correlation between a variable and a lag of this variable that is not explained by correlation at all low-order lags. Parameters in the ARIMA model(s) are estimated with the conditional least squares (CLS) method [29]. Since there are three parameters in an ARIMA model, different parameter combinations will lead to various results. The second step is to fit an optimum ARIMA model based on the Bayesian information criterion (BIC), for which the less BIC is, the better the model fits the data. Finally, the fitted model can be used to forecast the density of living snails and its confidential interval [30]. IBM SPSS 20.0 (IBM Corporation USA, http://www-01.ibm.com/software/analytics/spss/) was used for all the analysis.

## Results

### 1. Univariate analyses

**Fig 3:**
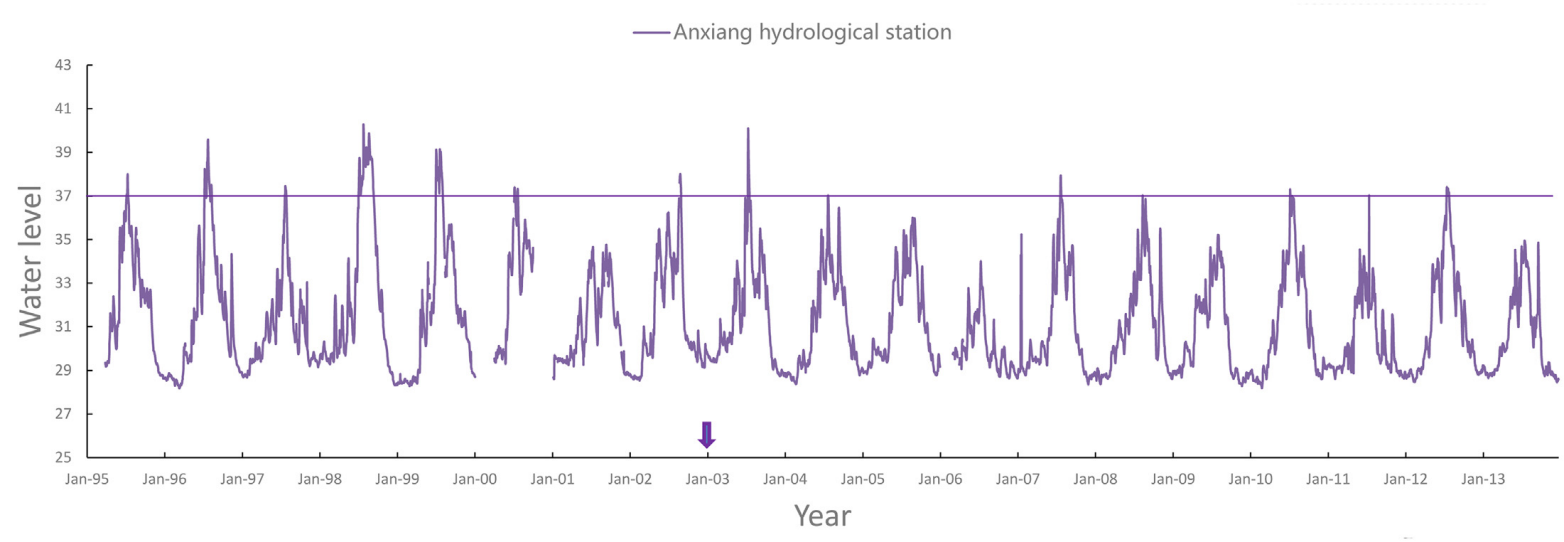
Plot of water level of Anxiang hydrological station.

Fig 3 shows the water levels at the Anxiang hydrological station from 1995 to 2013, with a warning water level for flooding of 37.0 m. June 1^st^ 2003 is the day that TGD began to impound. Before the year of 2003, 7 out of 9 years had a water level beyond the warning level at least once at this station compared with only 3 out of 10 years after the year of 2003 (2003 not included. Fig 3 also shows similar decreasing trends for the occurrence frequency of flooding and water level after 2003.

**Fig 4:**
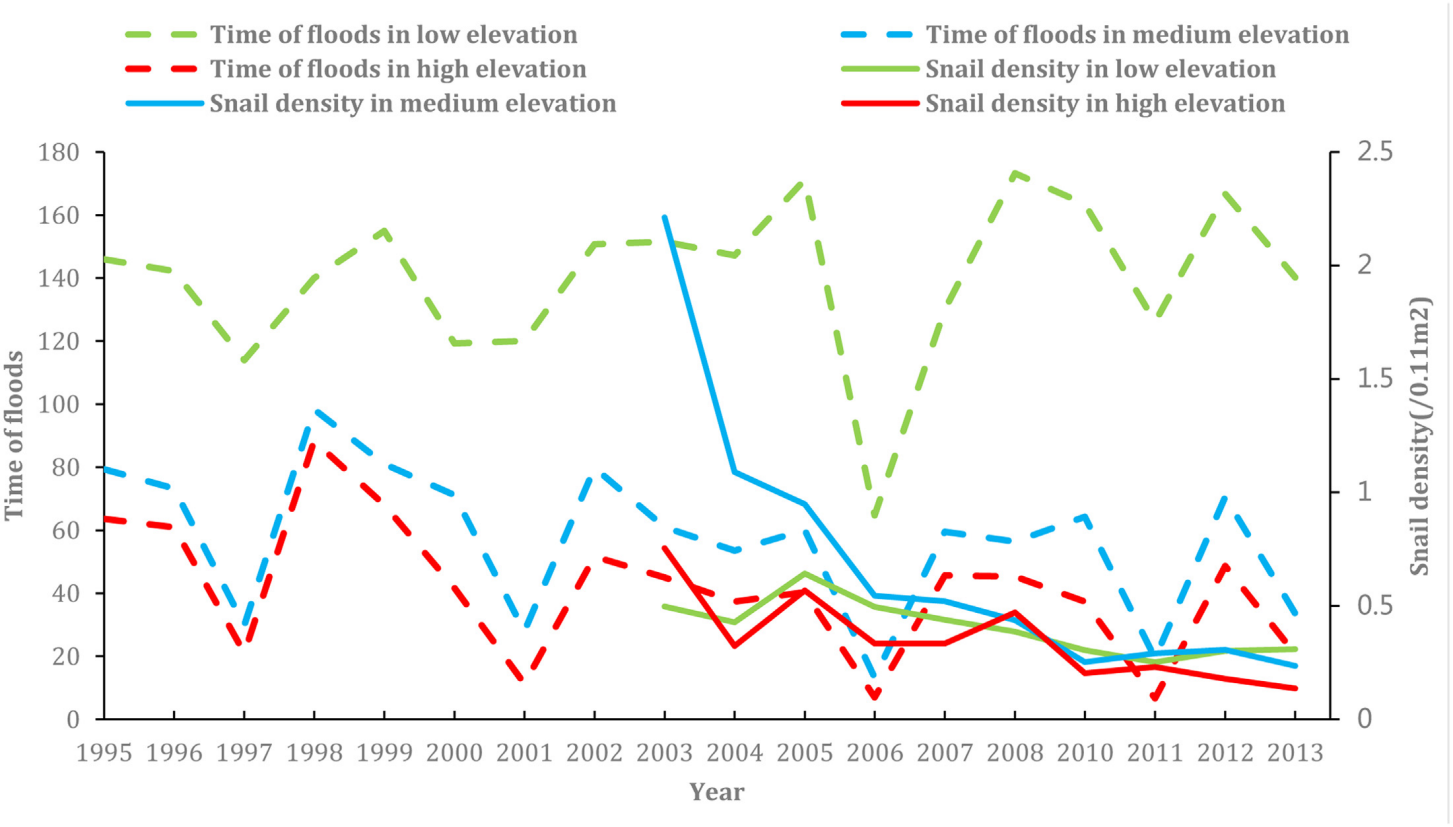
Number of flooding per year and density of living snails in different elevation areas.

Fig 4 shows the number of flooding per year and density of living snails in low, medium and high elevation areas. The data of 2009 were not included due to its too many outliers. The density of living snails was correlated with the water level a year before, which is caused by a lag effect of the water level. There were two severe droughts happened in 2006 and 2011.

The density of living snails in the high and medium elevation areas had a continuously decreasing trend from 2004 to 2014. In the low elevation area, the density of living snails had a similar trend between 2004 and 2012 but turned to increase after 2012. In the medium elevation areas, the density of living snails decreased sharply from 2004 to 2008 and stabilized after 2008.

Table 1 presents number of flooding per year before and after 2003 in 3 different elevations. After the construction of TGD, the number of flooding significantly decreased in the areas of both medium elevation (*t* = 4.519, *p*<0.000) and high elevation areas (*t* = 3.475, *p* = 0.001), but increased slightly in the low elevation area (*t* = -1.428, *p* = 0.158).

**Table 1:** Number of flooding per year before and after 2003 in different elevation areas.

	*Low elevation*		*Medium elevation*		*High elevation*	
	Before 2003	After 2003	Before 2003	After 2003	Before 2003	After 2003
Minimum	88	43	1	2	3	3
Maximum	166	199	118	130	91	54
Mean	135.9	144.4	67.56	47.58	50.83	31.88
Standard deviation	19.01	32.62	28.9589	27.96	24.84	16.35

### 2. Time-series analysis

ARIMA model was fitted to predict the density of living snails in the future in the 3 different elevation areas in the basis of historical time-series data. Autocorrelation graphs of the density of living snails indicated that the data were randomized and smooth. The parameter group (1,0,0) was preferred according to the BIC values of the density of living snails in the low, medium and high elevation areas (-4.12, -0.61 and -2.71).

**Fig 5:**
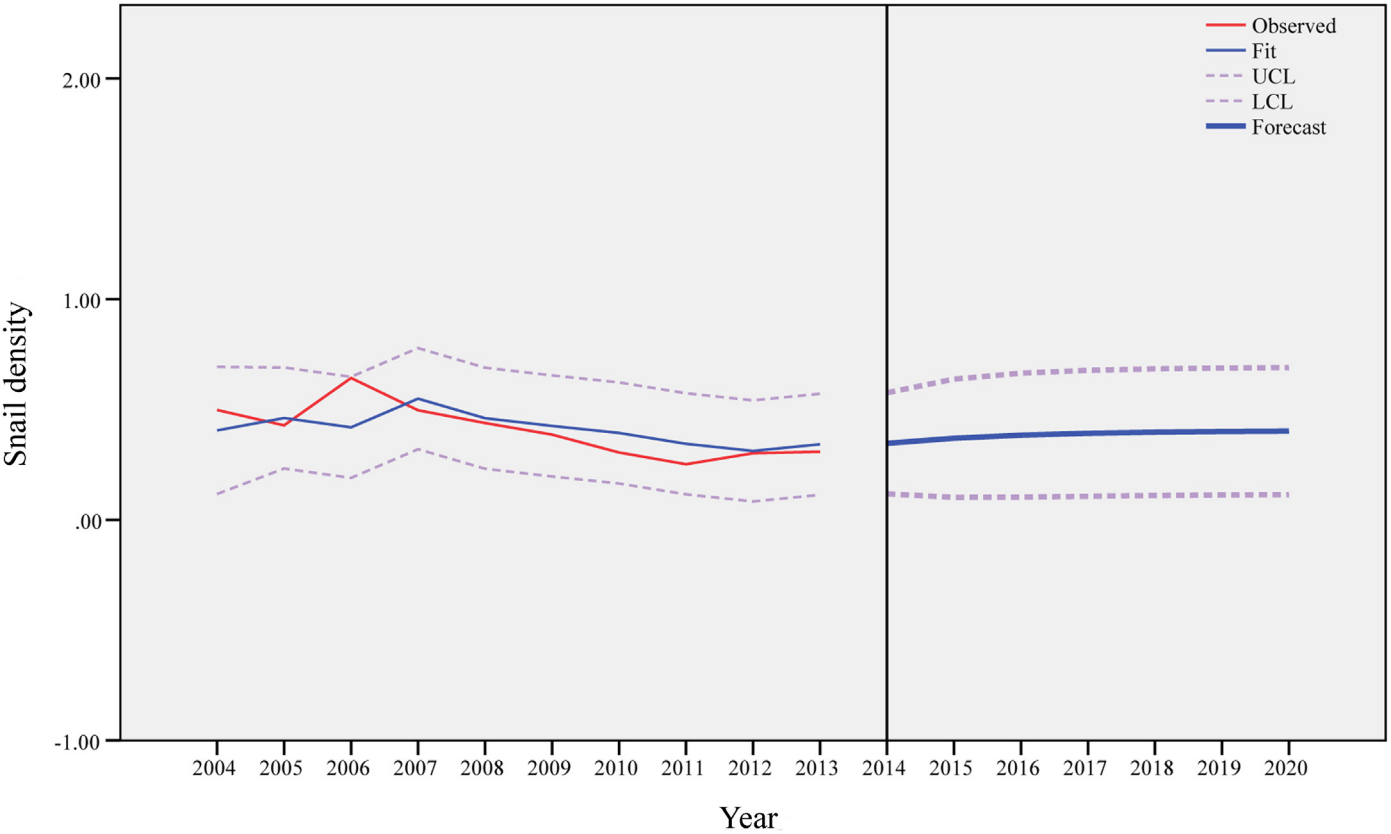
Observed and predicted density of living snails in the low elevation area.

Fig 5 shows the results of ARIMA (1,0,0) model in the low elevation area, which generates predicted values based on historical time-series data. The predicted value of the density of living snails in the low elevation area would increase from 0.37 /0.11 m^2^ in 2015 to 0.40/0.11 m^2^ in 2019 and after.

**Fig 6:**
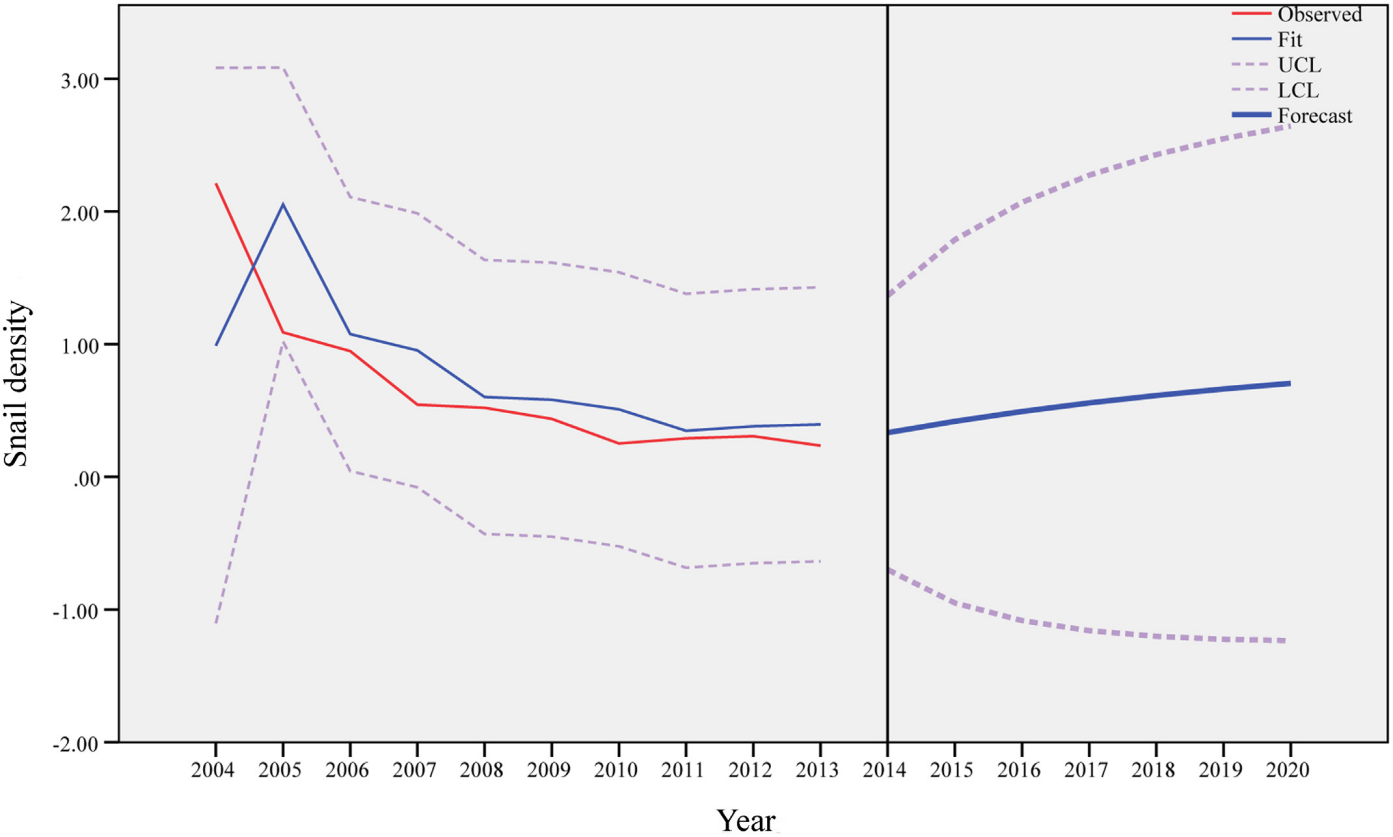
Observed and predicted density of living snails in the medium elevation area.

**Fig 7:**
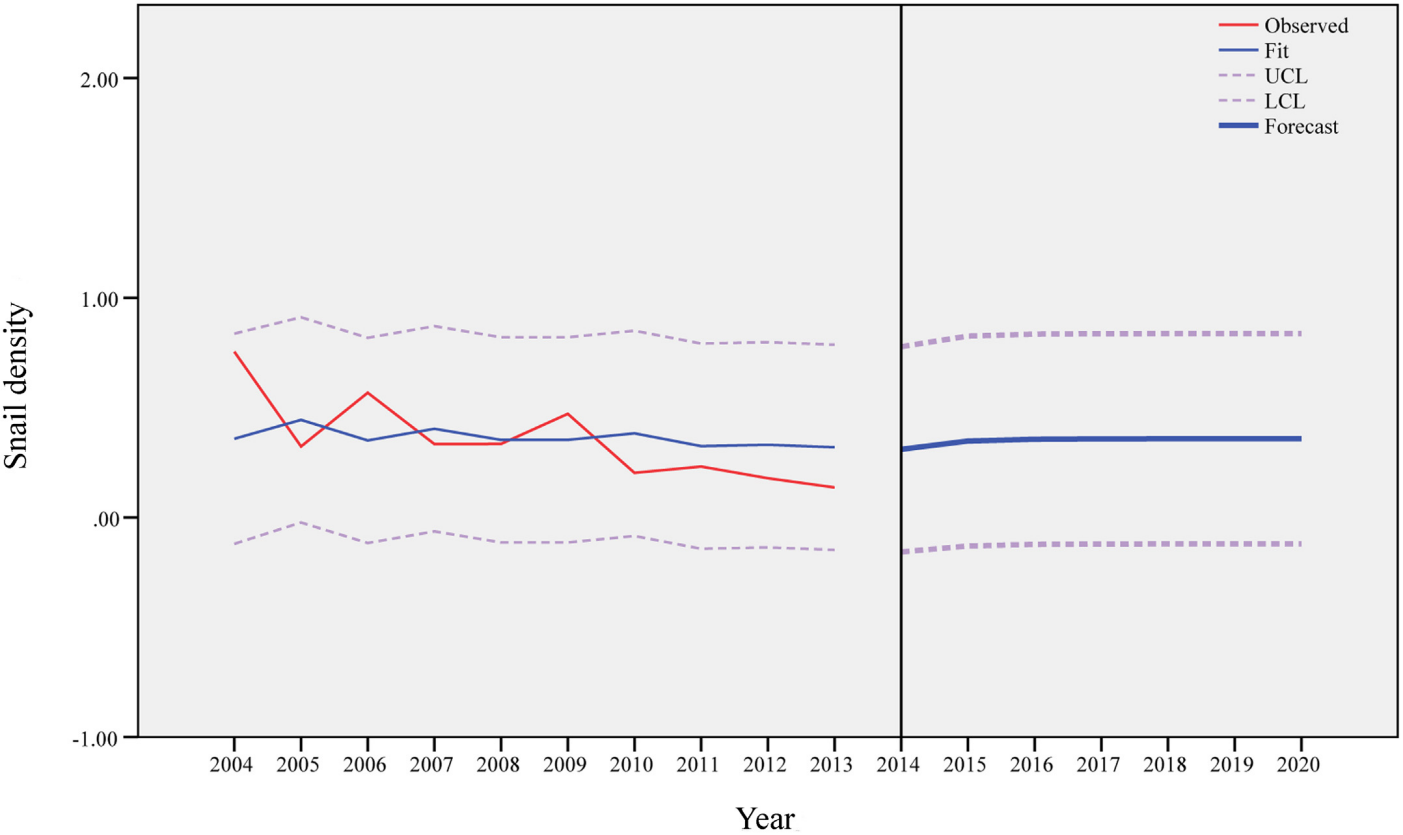
Observed and predicted density of living snails in the high elevation area.

Similarly, Fig 6 shows the results of ARIMA (1,0,0) model for the medium elevation area, and the predicted value of the density of living snails would increase from 0.42/0.11m^2^ in 2015 to 0.71/0.11m^2^ in 2020. Fig 7 presents the findings of ARIMA (1,0,0) model for the high elevation area, and the predicted values would not change markedly between 2015 and 2020.

## Discussion

After the impoundment of TGD in 2003, the dry season in Dongting Lake region was reported to arrive earlier and to be longer than before, and the water level was close to the lowest level in history for several times [31–34]. Anxiang County of Hunan Province is located in Dongting Lake region and at the downstream of TGD. Our results indicated that the water levels in all the elevation areas and number of flooding decreased after 2003 and that there was a severe drought in 2006.

Overall, there was a correlation between water level changing and density of living snails variation in all the elevations areas. The density of living snails in all elevations areas was decreasing after the TGD was built. The relationship between number of flooding per year and the density of living snails was more complicated. In the medium and high elevation areas, the density of living snails kept decreasing from 2003 to 2014. In low elevation area, however, the density of living snails decreased after 2003 first and turned to increase after 2011. The data of 2014 show that, the value of the density of living snails high in the low elevation area, low in the high elevation area and in between in the medium elevation area. Number of flooding per year decreased after 2003, so was the density of snails in the medium and high elevation areas. The association did not reach statistical significance in the low elevation area. *Oncomelania* is an amphibious snail, and its larva needs to live in water. When it grows into adult stage, it tends to inhabit in a humid region, like grass. Water is one of the necessary conditions for growth and reproduction of the snails that have to live in water or a wet place, and it is difficult to survive in the dry environment [35–37]. In the medium and high elevation areas, the reduced number of flooding per year might result in droughts in some months, which led to the decreased density of living snails. It might not be the case in the low elevation area. It is reported that the density of living snails in middle reaches of the Yangtze River including Dongting Lake Region is sharply reduced from 2003 to 2014 [38]. Another research finds that the density of living snails in Poyang Lake Region is declined after the impoundment of TGD [39]. The results in this study are consistent with the previous findings.

Droughts in this area are associated with the density of living snails. The severe drought in 2006 caused a decline of the density of living snails in 2007. The frequency of droughts in the medium and high elevation areas after 2003 was more than that before 2003, and as a consequence the density of living snails had a marked change. In the low elevation area however, the frequency of droughts after 2003 was less than that before 2003 and the decline of the density of living snails in this region was less obvious as compared with other areas.

Based on our prediction models, the snails would not disappear in the Dongting Lake region in near future. In the low elevation area, the density of living snails would increase slightly and then stabilize after the year of 2017. In the medium elevation region, the change of the density of living snails would be more obvious and would increase till the year of 2020. In the high elevation area, the density of living snails would remain stable after the year of 2015. Control of the snails would continue to be a challenge in the study area in the coming decade, and some measures, such as snail surveillance and fencing bovines on the marshland, need to continue to be implemented.

There are some limitations in this study. Firstly, data of the density of living snails were not available before 2003 and we were not able to make a comparison of the density of living snails before and after TGD was built. Secondly, only water level and elevation were studied while other factors such as climate conditions and plant types were not considered. However, we did not identify any important factors that had changed dramatically during the study period.

In conclusion, hydrology is an important determinant for the density of living snails. The density of living snails changed in different elevation areas, which was correlated with the variation of hydrology. TGD influenced the hydrology in the study areas and further changed the density of living snails. Based on the results from ARIMA models, we predicted that controlling snails would continue to be a challenge in the study area in the coming decade although TGD might lead to a reduction in the density of living snails in the region.

## References

[1] Zhou YB, Liang S and Jiang QW, et al Factors impacting on progress towards elimination of transmission of schistosomiasis japonica in China. Parasit & Vectors, 2012; 5: 275. 10.1186/1756-3305-5-275 PMC3519747 23206326

[2] Tang FY, Cheng YJ, Bso CJ, et al Spatio-Temporal Trends and Risk Factors for Shigella from 2001 to 2011 in Jiangsu Province, People's Republic of China. Plos one, 2014; 1(9): e83487. 10.1371/journal.pone.0083487 PMC3885411 24416167

[3] Li YS, Sleigh AC, Ross AG, et al Epidemiology of Schistosoma japonicum in China: morbidity and strategies for control in the Dongting Lake region. Int J Parasitol, 2000; 30(3): 273–81. 10719120 10.1016/s0020-7519(99)00201-5

[4] Zhou YB, Liang S, Chen GX, et al Spatial-temporal variations of Schistosoma japonicum distribution after an integrated national control strategy: a cohort in a marshland area of China. BMC Public Health, 2013; 13: 297 10.1186/1471-2458-13-297 23556428 PMC3621803

[5] Wu JY, Zhou YB, Li LH et al Identification of optimum scopes of environmental factors for snails using spatial analysis techniques in Dongting Lake Region, China. Parasit & Vectors, 2014; 7: 216. 10.1186/1756-3305-7-216 PMC4025561 24886456

[6] Zhang Z, Bergquist R, Chen D, et al Identification of parasite-host habitats in Anxiang county, Hunan Province, China based on multi-temporal China-Brazil earth resources satellite (CBERS) images. PLoS One, 2013; 8(7): e69447 10.1371/journal.pone.0069447 23922712 PMC3726693

[7] Zhou YB, Zheng HM and Jiang QW, et al A diagnostic challenge for schistosomiasis japonica in China: consequences on praziquantel-based morbidity control. Parasit & Vectors, 2011; 4: 194. 10.1186/1756-3305-4-194 PMC3195757 21981948

[8] Lin DD, Wu XH, Jiang QW, et al Strategic emphasis for research development of schistosomiasis control in China. Chin J Schisto Control, 2009; 21(1): 1–5.

[9] Zhou XN, Jiang QW, Guo JG, et al Road map for transmission interruption of schistosomiasis in China. Chin J Schisto Control, 2012; 24(1): 1–4. 22590853

[10] Zhou XN, Jiang QW, Sun LP, et al schistosomiasis control and surveillance in China. Chin J Schisto Control, 2005; 17(3): 161–165.

[11] Zhou XN, Jiang QW, Wang TP, et al Status and strategy for research development of schistosomiasis control in China. Chin J Schisto Control, 2005; 17(1): 1–3.

[12] Qiu J. Hydropower. Trouble on the Yangtze. Science,. 2012; 336(6079): 288–291. 10.1126/science.336.6079.288 22517834

[13] Xu XJ, Wei FH, Cai SX, et al Study on the risk factors of schistosomiasis transmission and control strategy in the Three Gorges Reservoir Areas. Chin J Schisto Control, 2004; 25(7): 559–63. 15308032

[14] Zhu HM, Xiang S, Yang K, et al Three Gorges Dam and its impact on the potential transmission of schistosomiasis in regions along the Yangtze River. Ecohealth, 2008; 5(2): 137–48. 10.1007/s10393-008-0168-y 18787917

[15] Zheng J, Gu XG, Xu YL, et al Relationship between the transmission of schistosomiasis japonica and the construction of the Three Gorge Reservoir. Acta Trop, 2002; 82(2): 147–156. 12020887 10.1016/s0001-706x(02)00046-3

[16] Seto EYW, Wu W, Liu HY, et al Impact of Changing Water Levels and Weather on Oncomelania hupensis hupensis Populations, the Snail Host of Schistosoma japonicum, Downstream of the Three Gorges Dam. EcoHealth, 2008; 5(2): 149–158. 10.1007/s10393-008-0169-x 18787918

[17] Wu JY, Zhou YB, Li LH, et al Identification of optimum scopes of environmental factors for snails using spatial analysis techniques in Dongting Lake Region, China. Parasites & vectors, 2014; 7(1): 216. 24886456 10.1186/1756-3305-7-216 PMC4025561

[18] Yao BD, Wang ZL, Zhang ZJ, et al Application of multi-temporal China-Brazil Earth Resources Satellite-02 data on surveillance of dynamic changes of water body of rivers and oncomelania snail habitats in Anxiang. Chin J Schisto Control, 2012; 24(02): 160–163+167. 22799159

[19] Zhou YB, Liang S, Jiang QW, et al An integrated strategy for transmission control of Schistosoma japonicum in a marshland area of China: findings from a five-year longitudinal survey and mathematical modeling. Am J Trop Med Hyg, 2011; 85(1): 83–88. 10.4269/ajtmh.2011.10-0574 21734130 PMC3122349

[20] Bowerman BL. Forecasting and Time Series: An applied approach. Belmont: Duxbury Press; 1993.

[21] Nsoesie EO, Mekaru SR, Ramakrishnan N, et al Modeling to predict cases of hantavirus pulmonary syndrome in Chile. PLoS Negl Trop Dis, 2014; 8(4): e2779 10.1371/journal.pntd.0002779 24763320 PMC3998931

[22] Gorge B. Time Series Analysis: Forecasting & Control. Pearson Education; 1994.

[23] Hou SY, Yan YP, Zhang ZY, et al Forecast of schistosomiasis endemic situation in the areas of "breaking dikes or opening sluice fro water store" in Dongting Lake using the time series analysis methods. Chin J Parasit Dis Con, 2004; 17(6): 38–40.

[24] Hou SY, Zhang ZY, Xu DZ, et al Application of time series analysis in the prediction of schistosomiasis prevalence in the areas of "breaking dikes or opening sluice for waterstore" in Dongting Lake. J Fourth Mil Med Univ, 2003; 24(24): 2297–2300. 15631742

[25] Hou XY, Zhang ZY, Xu DZ, et al Application of “time series analysis” in the predietion of sehistosomiasis prevalence in areas of “breaking dikes or opening sluice for water store” in Dongting Lake areas, China. Chin J Epidemiol, 2004; 25(10): 40–43. 15631742

[26] Chaudhuri S and Dutta D. Mann-Kendall trend of pollutants, temperature and humidity over an urban station of India with forecast verification using different ARIMA models. Environ Monit Assess, 2014; 186(8): 4719–42. 10.1007/s10661-014-3733-6 24705814

[27] Ramirez AP, Buitrago JI, Gonzalez JP, et al Frequency and tendency of malaria in Colombia, 1990 to 2011: a descriptive study. Malar J, 2014; 13: 202 10.1186/1475-2875-13-202 24885393 PMC4046009

[28] Grahn T. A conditional least squares approoach to bilinear time series estimation. Journal of Time Series Analysis, 1995; 16:509–529.

[29] Ho SL, Xie M, Gao TM. A comparative study of neural network and Box-Jenkins ARIMA modeling in time series prediction. Computers & Industrial Engineering, 2002; 42: 371–375.

[30] Galbraith J, Zinde-Walsh V. On the distributions of Augmented Dickey-Fuller statistics in processes with moving average components. Journal of Econometrics, 1999; 93:25–47.

[31] Zhang HJ and Guo JG. Impact of the Three Gorges Dam Construction on Transmission of schistosomiasis in the Reservoir Area. Chin J Parasitol Parasit Dis, 2006; 24 (03): 236–240. 17094632

[32] Zhang JH, Hu F, Guan CL, et al Impact of a Reservoir Project on schistosomiasis Transmission in Lake Region. Chin J Parasitol Parasit Dis, 2003; (01): 11–13. 12884579

[33] McManus DP, Gray DJ, Ross AG, et al schistosomiasis research in the dongting lake region and its impact on local and national treatment and control in China. PLoS Negl Trop Dis, 2011; 5(8): e1053 10.1371/journal.pntd.0001053 21912706 PMC3166040

[34] Zhang Z, Zhu R, Ward MP, et al Long-term impact of the World Bank Loan Project for schistosomiasis control: a comparison of the spatial distribution of schistosomiasis risk in China. PLoS Negl Trop Dis, 2012; 6(4): 1620. 10.1371/journal.pntd.0001620 PMC3328430 22530073

[35] Zhou XN, Zhang Y, Hong QB, Xu JD, Wang TP. Science on Oncomelania Snail. Sci Press, Beijing, China; 2005.

[36] Wang RB, Zheng J. Three Gorges Dam project and the transmission of schistosomiasis in china. Chin J Schisto Control, 2003; 15(01): 71–74.

[37] Wu CG, Zhou XN, Xiao BZ. The relatioship between changes of ecological environment after build of Three Gorges Dam and transmission of schistosomiasis. Foreigh Med Sci Parasit Dis, 2005; 32(5): 224–228.

[38] Zhu CF, Zeng QF, Li YY. Evaluation of the changes of snails and schistosomiasis in the middle reaches of the Yangtze River, Dongting Lake and Poyang Lake after the operation of Three Gorges project, in Academic annual meeting of the Chinese Water Conservancy Society in 2014. Tianjing, China.

[39] Chen HG, Zeng XJ, Lin DD. The changes of hydrological regime in Poyang Lake after runs of Three Gorges Project and its impact on prevalence of schistosomiasis in the lake region. Chin J Schisto Control, 2013; 25(5): 444–450. 24490349

